# Emerging Sensors Techniques and Technologies for Intelligent Environments

**DOI:** 10.3390/s22176427

**Published:** 2022-08-26

**Authors:** Ionut Anghel, Tudor Cioara

**Affiliations:** Computer Science Department, Technical University of Cluj-Napoca, Memorandumului 28, 400114 Cluj-Napoca, Romania

The trending techniques for managing indoor and outdoor intelligent environments rely heavily on data acquisition through a diversity of heterogeneous Internet of Things (IoT) devices and sensors. They process and analyze large and heterogeneous streams of data to extract knowledge and make reinforced decisions to adapt and optimize the interactions and enhance the users’ experience. On top, innovative services supported by sensor-based monitoring are developed to enable the intelligent management of smart cities, smart grids, ambient-assisted living, factories of the future, logistics chains, etc. These services benefit from the emerging technologies that can provide the needed infrastructure for their operation, such as cloud-based models, fog, edge computing, peer to peer, etc.

The “Emerging Sensors Techniques and Technologies for Intelligent Environments” Special Issue (SI) of the *Sensors* Journal aims to present a selection of state-of-the-art solutions to develop and manage intelligent environments. This SI features thirteen accepted articles covering various types of services such as energy efficiency and demand response (DR) [1,2]; wireless-sensor-network optimization [3]; natural language comprehension and ambient intelligence [4,5]; water, air, and heat management [6,7,8]; smart transportation and intelligent vehicles [9,10]; personal data security [11]; smart farming [12]; and the detection of plant diseases [13]. Each manuscript has been reviewed by independent experts in the respective article’s domain and has been through multiple stages of peer-review.

In [1], the authors present an implementation of decentralized DR programs in a smart grid using a public blockchain focused on energy data privacy using Zero-knowledge proofs (ZKP). The smart meters gather the prosumer energy consumption. The information is maintained privately by storing the associated zero-knowledge-proof generated by the prosumer in the blockchain. Validation functions implemented in smart contracts use the proof to check for potential deviations from the DR requests and settle the prosumer’s activity in the program. In [2], the prediction of energy consumption to enable the participation of blocks of buildings in DR is addressed, focusing on the analysis of the detection of outliers using machine learning (ML) techniques. A numerical algorithm is proposed to differentiate between natural energy peaks and outliers, and the impact on the energy baseline for the DR computation is discussed.

The authors of [3] propose an energy-efficient routing algorithm that provides end-to-end reliability in multi-hop wireless sensor networks. It uses implicit acknowledgment to offer reliability and connectivity with low latency and fault tolerance in linear wireless sensor networks. The results show that the algorithm decreases energy consumption and minimizes delays while increasing network reliability and connectivity. In [4], a customized capsule neural network architecture is defined to perform intent detection and slot filling to handle utterances containing various levels of complexity. The authors address the problem of natural language comprehension in intelligent environments using a home assistant in the Romanian language. A solution for monitoring the activities of daily living and identifying human routines is defined in [5]. A Markov model is used to construct the daily routines. To measure the similarity between the day-to-day monitored activities and routines, the authors use the entropy rate and cosine functions. The techniques were integrated in a distributed monitoring system that uses IoT beacons and trilateration techniques for monitoring the activities of older adults.

In [6], the authors propose a smart sensor-based federated learning solution for water pollution management. An optimal scheduler is proposed to manage the real-time data being validated using data-intensive experiments with water quality data. The problem of data centers’ energy efficiency is addressed in [7] considering the heat reuse in the case of a distributed data center that features the servers’ installation in residential homes, allowing them to be used as a primary source of heat. A workload-scheduling solution is defined using a constraint-satisfaction model to optimally allocate workloads on servers to reach and maintain the desired home temperature setpoints. A mathematical model derived from thermodynamic laws is calibrated with monitored data and is used together with an ML model to predict the workload to be executed by a server to reach a desired ambient temperature setpoint. In [8], the focus is on predicting behaviors regarding the air quality index using ML for pollution management in smart cities. Three ML algorithms were studied and evaluated based on their prediction errors.

Article [9] addresses the design, development, and configuration of a laser system that can be used to obtain the 3D profiles of vehicles and more precise information about the states of roads. The criteria to design such systems are detailed and a complete laser system implemented for vehicle detection and classification is presented. In [10], the authors propose an adapted trip-chaining method to reconstruct incomplete multi-modal journeys for public transport systems. A space-time characterization of the overall operation of the transport networks is constructed followed by mobility patterns between zones and average traversed distances, travel times, and operation speeds.

In [11], a method to securely manage personal information in IoT devices that complies with the General Data Protection Regulation is introduced. The lifecycle of personal information in IoT application services is detailed, and a method to securely manage personal information is presented. Article [12] presents an edge technology capable of handling the collection, analysis, prediction, and detection of heterogeneous data in the context of strawberry farming. The platform integrates and manages IoT devices to analyze environmental and crop information to mitigate a problematic disease and assess its impact on the environment. In [13], the authors include a study and review of the current advances in plant-disease detection using IoT remote sensing. The study identifies the current solutions and development directions and provides a comparative evaluation of the results involving different plants’ disease-detection and remote-sensing infrastructures.

The guest editors of this SI would like to thank all the authors who have submitted their work to the *Sensors* journal and to the “Emerging Sensors Techniques and Technologies for Intelligent Environments” SI, all the reviewers for their hard work during the review process, and the editors of *Sensors* for their kind and continuous support. We hope that this SI and the thirteen published articles within will help researchers to understand the state-of-the-art with respect to sensors, techniques, and technologies for intelligent environments and that it will help generate further research within the targeted domain.
